# Genetic Analysis of Major Carcass Traits of Korean Hanwoo Males Raised for Thirty Months

**DOI:** 10.3390/ani11061792

**Published:** 2021-06-15

**Authors:** Mahboob Alam, Soo Hyun Lee, Do Hyun Lee, Chungil Cho, Mi Na Park

**Affiliations:** 1 Animal Breeding and Genetics Division, National Institute of Animal Science, Cheonan-si 31000, Korea; mahboob@korea.kr (M.A.); lhyungm@korea.kr (S.H.L.); ldh9462@korea.kr (D.H.L.); 2 Hanwoo Genetic Improvement Center, NongHyup Agribusiness Group Inc., Seosan-si 31948, Korea; blup82@nonghyup.com

**Keywords:** heritability, genetic correlation, animal model, carcass weight, eye-muscle area, backfat thickness, marbling score, 30-month production period, Korean Hanwoo cattle

## Abstract

**Simple Summary:**

Generally, Korean Hanwoo males produced under a 24-month production cycle (PROD24) are evaluated as a part of the progeny test program. However, there is little information on other males outside the PROD24, such as those raised under a 30-month production cycle (PROD30) for higher profits. Therefore, we investigated PROD30 males for important carcass traits (carcass weight, eye muscle area, backfat thickness, and marbling score) using a reasonably large dataset to understand their genetic merit. To do so, we estimated the genetic parameters of traits using animal model. Our analysis revealed moderate to high heritability values for the studied traits. The marbling score was found to be highly heritable at 0.56. The genetic correlation between traits was mostly moderate to low, and the backfat thickness was poorly correlated with the marbling score. These results are consistent with many previous reports on PROD24. Our study suggests that PROD30 and PROD24 males might have somewhat similar genetic potential, as well as similar genetic backgrounds. Thus, it could be concluded that there is further scope for PROD30 males to improve Hanwoo males’ overall prediction accuracy, especially under a genomic selection program, together with PROD24 males.

**Abstract:**

Understanding animals’ genetic potential for carcass traits is the key to genetic improvements in any beef cattle. In this study, we investigated the genetic merits of carcass traits using Hanwoo males raised in a 30-month production system (PROD30). We achieved this using a dataset comprising 6092 Hanwoo males born between 2005 and 2017 and measures of four carcass traits (carcass weight, CWT; eye muscle area, EMA; backfat thickness, BFT; and marbling score, MS). Genetic parameters were estimated using a multiple-trait animal model through the AIREMLF90 program. According to the multiple-trait model, the h^2^ of CWT, EMA, BFT, and MS were 0.35 ± 0.04, 0.43 ± 0.05, 0.48 ± 0.05, and 0.56 ± 0.05, respectively. The strongest genetic correlation (r_g_) was obtained between CWT and EMA (0.49 ± 0.07), whereas it was negligible between CWT and BFT. EMA and MS were also moderately correlated, whereas there was a relatively low negative correlation between EMA and BFT (−0.26 ± 0.08). Our study revealed a consistent indirect genetic improvement in animals from 2005 onwards. Although Hanwoo improvement has mainly focused on males under a 24-month production cycle, we observed PROD30 males to have somewhat similar genetic potential. Our results provide useful insights into the genetic merits of PROD30 males for the first time, which may facilitate future studies on them and their integration into the Hanwoo National Evaluation for genomic selection.

## 1. Introduction

Korean Hanwoo cattle are the most prized source of beef in Korea due to their high marbling and palatability [1,2]. Decades-long planned breeding and evaluation systems have paved the way for significant production improvements in Hanwoo cattle [3,4]. In Korea, a national Progeny Test Program (PGT) is also at the center of the proven bull production system. The PGT includes progenies from two sources, such as those raised in the test station for 24 months (PROD24) and the others raised in various breeding farms for 30 months (PROD30). Farmers’ particular choice for the extended raising period is related to higher marbling scores (MS) and greater profits from animals. However, the national evaluation of Hanwoo males for carcass traits using PROD24 instead of PROD30 as the primary breeding objective was deemed a logical choice under the PGT due to the high cost of production. On the other hand, the existing proven bull selection process, based on PROD24, also prohibits PROD30 males from contributing directly to proven-bull evaluation, as the bull evaluation ends before PROD30 data become available. This resulted in a lower number of progenies for testing per young candidate bulls over the years.

The Korean National Hanwoo improvement policy has mainly focused on the PROD24 system since the 1980s. However, Hanwoo cattle face particular challenges from a population genetics standpoint due to their limited gene pool. At the same time, the Hanwoo breeding policy does not encourage the inclusion of foreign genetic resources due to the strict pure breeding strategy. Thus, it is crucial that all potential sources of genetic variation within existing populations, such as PROD30 males, are exploited properly. Although the PGT program showed significant successes in improving carcass performance over the years [5,6], the future production of the best-performing bulls might encounter additional challenges. The PGT recently adopted a policy for increasing the number of selected proven bulls per year. This raises further concerns as the PGT program has a limited capacity for progeny rearing. Given this restriction, the number of progenies to be tested per year per candidate bull has reduced further, which is undesirable in any proven bull selection program and could lower the accuracy of future proven bulls. In this regard, the inclusion of PROD30 males in the national evaluation through a proper genetic evaluation method could help increase the number of progeny records per bull and contribute to the accuracy of evaluation in the long run. The recently adopted single-step genomic BLUP (ssGBLUP)-based genomic selection (GS) in PGT [7,8,9] could provide an opportunity in this regard as it would allow all animals to participate in the evaluation process of animals. Thus, the currently implemented ssGBLUP-based animal evaluation coupled with PROD30 males could be a solution to the current PGT limitations. To do so, the genetic potential of PROD30 males for carcass traits demands investigation at first.

The genetic potential for economic traits is usually assessed by population genetic parameters such as heritability and genetic correlation estimates. More accurate knowledge of these parameters leads to better genetic evaluation and breeding programs with maximum selection response [10]. Heritability is the most significant genetic parameter that expresses the degree of correspondence between phenotypic values and breeding values [11]. The other parameter, the genetic correlation between traits, also indicates the simultaneous change in one trait with respect to the selection of another trait. Furthermore, the selection of animals is shown to be most effective when the relationships among the selected traits are accounted for [12]. On the other hand, genetic parameters are known to be specific to a breed or a population or to be environment-specific, and factors such as selection could change their population potentials [11,13]. Therefore, the nature of the inheritance of carcass traits must be studied explicitly in any given population. 

Previously, the genetic parameters for the PROD24 population have been reported in numerous studies [1,5,9,14,15,16,17]. In these reports, carcass weight (CWT) and eye muscle area (EMA) are generally found as being moderately heritable, such as between 0.298 and 0.36 and between 0.27 and 0.44, respectively [5,9,14]. The backfat thickness (BFT) is also reported as being highly heritable in Hanwoo males, i.e., h^2^: 0.46–0.51 [5,9,14,18]. Similarly, MS is found to be highly heritable (h^2^: 0.48–0.63) in various reports [5,9,14,18,19]. Previous reports generally suggest low and positive genetic correlations between CWT, BFT, and MS [5,9,14]. The BFT and EMA association is also found as being low and oppositely correlated [5,9,14]. The MS and BFT relationship varied between slightly negative and slightly positive among reports [5,9,14]. There are no reports available on PROD30 males under PGT. However, a study on 7991 commercial Hanwoo cattle slaughtered at 30-month age found genetic parameter estimates within the range of other reports as shown above [20]. The purpose of this study was to investigate the heritability and genetic correlations among traits in PROD30 males. We believe that this would provide a better understanding of their genetic merit and expand further possibilities for their genetic exploitation, especially through the genomic selection of animals.

## 2. Materials and Methods

### 2.1. Animals and Phenotypes

The study population comprised 6092 Hanwoo males in the PROD30 population that were born between 2005 and 2017 in various designated Hanwoo breeding farms across Korea. The data on carcass phenotypes were provided by the Hanwoo Improvement Center, Seosan, Korea. All the males were classified into three groups based on their purposes of use (Table 1). The first group of males (G1) was comprised of those produced under the performance-test program but not selected for progeny testing. The second group of males (G2) indicated those produced under the progeny test program but not used in the proven bull evaluation. Finally, the last male group (G3) was comprised of males produced in another special program, wherein their semen was produced at an earlier age, i.e., about 12 months, and preserved for future breeding of females once these males’ breeding values for 30-month post-slaughter carcass traits were found to be superior. Each group of males was produced in two batches in a year. All production batches were mostly non-overlapping, indicating distinct seasonal influences on their growth. Moreover, the locations in which they were raised differed: G1 and G3 males were raised in the Hanwoo Improvement Center and a few other designated centers across the country, while G2 males were mostly raised in designated Hanwoo breeding farms across the country. All males were slaughtered between the age of 27 and 33 months. The slaughter locations of G1 and G3 males were mostly the same for a particular raising station or farm. However, the G2 males were slaughtered in random locations due to farm-specific preferences. After slaughtering males by following the guidelines of the Korea Animal Improvement Association (KAIA), four carcass measurements—carcass weight (CWT; kg), eye-muscle area (EMA; cm^2^), backfat thickness (BFT; mm), and marbling score (MS; 1–9)—were recorded. An MS value of 1 indicated the poorest degree of marbling, whereas a score of 9 indicated the greatest degree of marbling in beef. The feeding of PROD30 males was variable across farms and ad libitum in nature, especially during the last few months before slaughter when they were mostly provided with high-energy diets to increase MS. It is important to note that this feeding practice was somewhat different from commonly used feeding practices for PROD24 males [14], where the feeding practice is generally as per recommendations.

### 2.2. Animal Pedigree

A pedigree related to the studied males was obtained from KAIA. The pedigree was composed of 25,032 animals, where the longest ancestral path was traced up to 14 generations, with about 38.13% of those being inbred. Moreover, a significant proportion (96%) of the inbred animals had lower inbreeding rates (0–5%). The highest inbreeding coefficient was 0.26. However, the average inbreeding coefficient was observed as being as low as 0.01 in the pedigree. The software package CFC 1.0 [21] was used to investigate pedigree structures and inbreeding coefficients in this study.

### 2.3. Statistical Analysis

#### 2.3.1. Factor Selection and Development of the Statistical Model

We selected appropriate fixed and random effects for the genetic analyses of traits (Tables S1–S4). A list of factors (i.e., animal type, batch number, birth year, birth season, raising location, slaughterhouse, slaughter date, and slaughter age) were tested for statistical significance. Each factor’s significance was determined in R using the ‘glm’ (generalized linear model) function [22]. Among these factors, animal type, batch number, birth year, birth season, and raising location were tested as either single or combined effects, considering their interaction effects (Tables S1–S4). Animal type, which was treated as a fixed effect, accounted for the systematic differences between animal groups. Following previous reports [9,14], batch-number was fitted as an indicator of fixed effects from the year and season of birth. We also modeled the direct effect for birth year and birth season (winter, spring, summer, and autumn) to assess the batch number effect. The fixed effect of raising-location accounted for any systematic, environmental, or management influences within the population.

Similarly, slaughterhouse and slaughter date were tested as either fixed or random effects and with and without interaction terms. The slaughter age factor was fitted as a fixed covariate with all of the models. Finally, the Akaike information criterion (AIC) [23] and residual sum of squares (RSS) from all models were compared to determine the model with best fit based on the model with the lowest AIC and RSS. The best fit model comprised one compound fixed effect (animal type, birth year, birth season, and raising location), a compound random effect (slaughterhouse and slaughter-date), and a covariate effect (slaughter-age); each of these terms was selected for further animal model analysis.

#### 2.3.2. Estimation of (co)Variances, Genetic Parameters, and Genetic Trends

The (co)variance components of carcass traits were estimated through a multiple-trait animal model using the AIREMLF90 software package [24]. The final animal model included a combined fixed effect (TYSL), a combined random effect (SHD), a fixed covariate of slaughter age (SAGE), and a random additive genetic effect. The composite fixed effect, TYSL, was constructed by combining the animal type, birth year, birth season, and raising location effects. SHD was formed by merging slaughterhouse and slaughter-date effects. The matrix notation of the mixed model equation for the analysis was as follows:

(1) y=Xb+Za+Wu+e

where *y* is the vector related to the observations of carcass traits, *b* is the vector related to the fixed effects of TYSL and SAGE, *a* is the vector related to the random additive genetic effects of animals, u is the vector related to the systematic random effect of SHD, and *e* is the vector related to the random residuals. X, Z, and W are design matrices relating the observations to the model’s corresponding effects. Our assumed (co)variance matrices for random and residual effects were Var (a) = $G_{0}⊗A$, Var (u) = $R_{0}⊗I$**,** and Var (e) = $R_{0}⊗I$, where $G_{0}$, A, $R_{0},$ and I are the additive genetic (co)variance matrix between traits, the numerator relationship matrix, the residual (co)variance matrix between traits, and the identity matrix, respectively.

The total phenotypic variance ($\sigma_{p}^{2}$) was calculated as $\sigma_{p}^{2}=\sigma_{a}^{2}+\sigma_{u}^{2}+\sigma_{e}^{2}$, where the $\sigma_{a}^{2}$, $\sigma_{u}^{2}$ and $\sigma_{e}^{2}$ parameters are the additive genetic variance, the random variance of SHD, and the random variance of residuals, respectively. Trait heritability (h^2^) was obtained by the equation h^2^ = $\sigma_{a}^{2}$/$\sigma_{p}^{2}$. The genetic correlation (r_g_) between two traits was estimated as

(2) $$r_{g}=\frac{\sigma_{a_{1}a_{2}}}{\sqrt{\sigma_{a_{1}}^{2}\times \sigma_{a_{2}}^{2}}}$$

where $\sigma_{a_{1}}^{2}$ and $\sigma_{a_{2}}^{2}$ are the genetic variance estimates of traits 1 and 2, respectively, and $\sigma_{a_{1}a_{2}}$ is the genetic covariance between two traits. Similarly, we also estimated the phenotypic correlation (r_p_) between traits using the phenotypic variance and the covariance of two traits. Note that phenotypic correlation is provided for completeness only. We obtained the approximated standard error (SE) of genetic parameter estimates from (co)variance components using the appropriate AIREMLF90 option (‘se_covar_function’), which uses a Monte Carlo method for the computation of SE, as suggested by Houle and Meyer [25]. The genetic coefficient of variation (CV_g_) was also calculated as the ratio of a trait’s genetic standard deviation ($\sigma_{a}$) and its mean, according to Houle [26]. We also obtained the genetic trend in traits using the average estimated breeding value (EBV) of animals based on their birth year.

## 3. Results

### 3.1. Descriptive Statistics for Phenotypes

Table 2 presents a descriptive summary of CWT, EMA, BFT, and MS in Hanwoo males. Our observed mean for CWT was 447.00 ± 48.24 kg. For EMA, the average value was 93.75 ± 11.31 cm^2^. For BFT, the observed phenotypes ranged between 1 and 36 mm, with a CV estimate of 36.93%. In Hanwoo males, the highest phenotypic variability was found in MS (38.38%). However, the phenotypic variability was generally lower in CWT and EMA traits.

### 3.2. Genetic Parameter Estimates Using a Multiple-Trait Animal Model

Table 3 lists the (co)variance component estimates and genetic coefficient of variation estimates, while Table 4 presents the h^2^ and correlation estimates between traits using the multiple-trait model analysis. We classified all the h^2^ values into three categories: low (0.00 to 0.20), medium (0.21 to 0.40), and high (0.41 to 1.0), according to Bailey [27]. Both MS and BFT were highly heritable traits, with h^2^ values of 0.56 ± 0.05 and 0.48 ± 0.05, respectively. However, for CWT and EMA, the h^2^ values were mostly moderate, at 0.35 ± 0.04 and 0.43 ± 0.05, respectively. The CV_g_ of 27% in MS was the highest value among all four traits. The lowest genetic variation was found in CWT in our study.

Conversely, the r_g_ estimate indicated a moderate genetic correlation of 0.49 ± 0.07 between CWT and EMA, which was the highest correlation among all pairs of traits. This result suggests that the selection of CWT could lead to improved EMA and vice versa. The strength of association between EMA and MS was moderate and positive (0.48 ± 0.07). Between CWT and MS, the r_g_ was weakly positive (0.23 ± 0.08). However, the r_g_ value was found to be weakly negative between EMA and BFT (−0.26 ± 0.08), while it was very weakly negative between BFT and MS (−0.09 ± 0.08). We also observed an association between CWT and BFT that was statistically not different from zero.

### 3.3. Genetic Trends of Carcass Traits in Hanwoo Males

**Figure 1:**
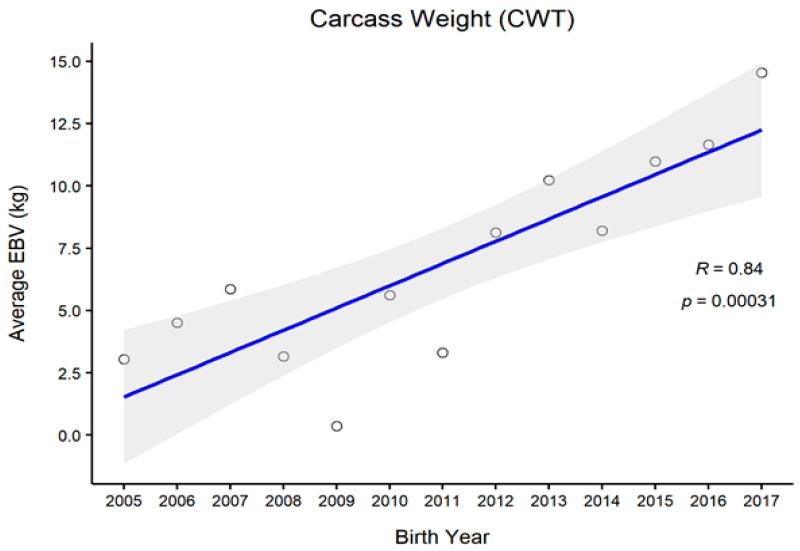
The genetic trend of estimated breeding values (EBVs) for the carcass weight of Hanwoo males from the 30-month production system (R, Pearson’s correlation coefficient; *p*, the *p*-value of correlation coefficients).

**Figure 2:**
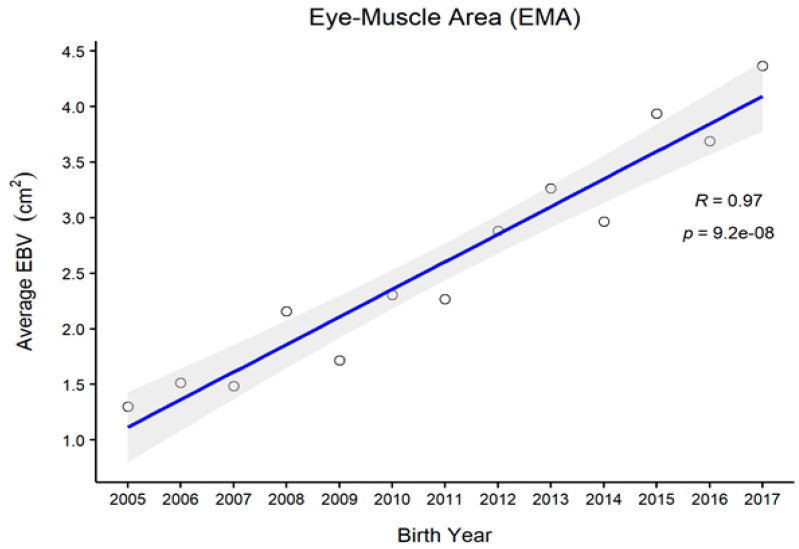
The genetic trend of estimated breeding values (EBVs) of the eye muscle area of Hanwoo males from the 30-month production system (R, Pearson’s correlation coefficient; *p*, the *p*-value of correlation coefficients).

**Figure 3:**
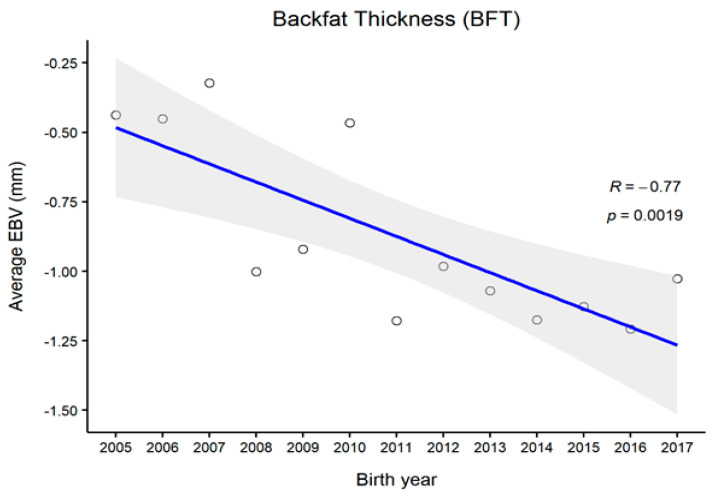
The genetic trend of estimated breeding values (EBVs) of backfat thickness of Hanwoo males from the 30-month production system (R, Pearson’s correlation coefficient; *p*, the *p*-value of correlation coefficients).

**Figure 4:**
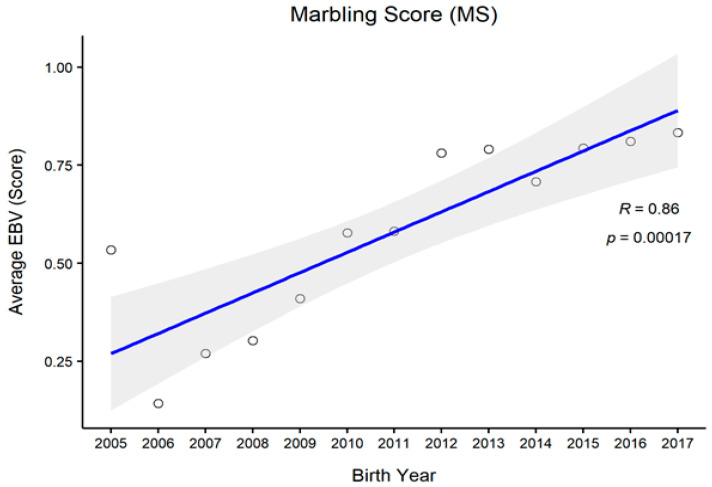
The genetic trend of estimated breeding values (EBVs) of marbling score of Hanwoo males from the 30-month production system (R, Pearson’s correlation coefficient; *p*, the *p*-value of correlation coefficients).

Figure 1, Figure 2, Figure 3 and Figure 4 represent the genetic trends of CWT, EMA, BFT, and MS. There was an overall EBV improvement in most of the traits from 2005 onwards. Despite some inconsistencies, the overall results indicate no noticeable decline in genetic trends. We observed an increase in the EBV for CWT from 2.5 to 15.0 kg, with a Pearson’s correlation (R) of 0.84 (*p* < 0.001). Similarly, EMA rose from 1.2 cm^2^ to 4.5 cm^2^ (R = 0.97, *p* < 0.001). However, the EBV of BFT reduced from −0.50 to −0.90 mm (R = −0.77, *p* < 0.001). Although an overall decline was obvious in BFT-EBVs, the changes were somewhat inconsistent over time. We observed desirable changes in the EBV of MS over the study period, with values going from 0.5 to 0.75 (R = 0.86; *p* < 0.001).

## 4. Discussion

This study found all carcass traits to be moderate to highly heritable in PROD30 males. Our estimated heritability for CWT was consistent with the value of Park et al. [9], who reported a h^2^ of 0.35 using males from the PROD24 population. Another recent Hanwoo study reported a h^2^ of 0.36 [19], which is similar to our estimate. Results from other studies were also similar to ours [15,16,18,28]. Nevertheless, some studies have reported slightly lower h^2^ values of 0.29 ± 0.04 and 0.30 ± 0.04 in Hanwoo cattle [5,14]. In comparison to other cattle breeds, the CWT h^2^ values we found were somewhat similar to Simmental [29] and a few other breeds [30,31,32,33]. For the h^2^ of EMA, we also found agreement with Park et al. [9], who reported a value of 0.44. Choi et al. [14] reported a slightly lower h^2^ value (0.38 ± 0.05) in Hanwoo steers. However, other Hanwoo reports were generally consistent with our report [5,16,18,34]. We also verified our moderate h^2^ estimate within the range of other breeds such as Nelore cattle (0.29 ± 0.07 to 0.33 ± 0.03), Japanese Brown cattle (0.29), Australian Angus and Hereford bulls (0.26–0.38), Japanese Black steers (0.43 ± 0.06), and Simmental bulls (0.26 ± 0.05 to 0.46 ± 0.05) [31,35,36,37,38,39,40]. Our results also demonstrate BFT to be a highly heritable trait. These values were consistent with many other Hanwoo reports as well [5,9,14,16,18,19]. However, we also noticed some differences in BFT estimates with other Hanwoo reports. Some reported slightly lower values [17,34,41]. Our findings are comparable with values reported in the literature for some other breeds [35,36,42]. Additionally, the current BFT h^2^ values appeared to be somewhat intermediate compared to other breeds, for which moderate to high values of h^2^ from 0.35 ± 0.05 to 0.58 were reported [37,38]. MS, which is the most valued trait in Hanwoo cattle, also showed the highest h^2^ estimate in this study. The finding of Park et al. [9] was in strong agreement with our estimate. Similarly, the current estimate appeared to be in line with some earlier reports based on ultrasonic or post-slaughter carcass data, where h^2^ values ranging from 0.48 to 0.54 were reported [15,18,41]. Our estimate was also similar to those reported in many other reports on other cattle such as 0.48 ± 0.03 to −0.59 ± 0.06 [31,38,43]. Among the few discrepancies in Hanwoo cattle, the reported h^2^ values by Shin et al. [19] and Choi et al. [14] were slightly higher than ours, with h^2^ values of 0.58 and 0.62 ± 0.07, respectively.

This study revealed a wide range of genetic correlation estimates between traits. We found both CWT and EMA to be moderately and positively correlated traits in PROD30 males. This could be due to the pleiotropy wherein a gene or a set of genes influences two traits and results in the genetic correlation between these traits [44]. This correlation estimate is found to be close to those of Park et al. [9] and Choi et al. [14], who reported values between 0.52 ± 0.08 and 0.55 using similar traits with PROD24 males. A similar positive correlation was found in other Hanwoo studies [18,45]. Several other breeds, such as Angus, Brahman, Nelore, and other crossbred cattle, also demonstrated agreement with the correlation we found between CWT and EMA [40,42,46,47,48]. Similarly, the non-significant association between CWT and BFT that we found is in agreement with results from other Hanwoo cattle studies [9,18,45]. However, the estimate was moderately low in another breed but higher than our estimate [49]. For CWT and MS, we found somewhat similar weak correlations (from 0.17 ± 0.11 to 0.30) in Hanwoo cattle reports and reports on other breeds [9,14,49]. The evidence of a negative correlation between EMA and BFT has been reported frequently, with r_g_ values ranging from −0.09 to −0.45 for Hanwoo cattle [34,41,50]. Studies on Brahman [47] and other crossbreeds [42] provided either a positive or absence of relationship between these traits, indicating partial support for our reported values. This negative correlation estimated between EMA and BFT in Hanwoo males suggests that selection for EMA or against BFT might indirectly improve the other trait. Additionally, the negative correlation in our study indicates their opposite direction of selection in Hanwoo cattle that is in practice over generations. In this regard, the importance of selection directions can be seen in a recent Brazilian study, which reported an undesirable positive relationship between EMA and BFT traits (0.53 ± 0.08) as a result of positive selection for both EMA and MS while keeping BFT at a constant level [51]. However, with regard to the association between EMA and MS, we found similar disagreements from recent reports of Park et al. [9] and Choi et al. [14], where the reported values were consistently lower (from 0.25 to 0.30). Such disagreement could be due to the difference in the studied population, where they all studied PROD24 males. Our further investigation shows that the overall variability in MS was relatively lower in our study (CV: 38.38%) than their reports (CV: 49.67–51.1%). This reduced MS variability in PROD30 males, which could be due to the farmers’ special feeding practices in the last few months before slaughter for gaining higher MS grades from males, might have affected the overall (co)variance structure between the traits towards a higher correlation. The absence of an association between BFT and MS was also consistent (r_g_ = 0.01–0.04) with findings in other breeds such as Brahman and Angus [49,52,53]. These traits also tend to be variably correlated (r_g_ = from −0.19 to 0.66 ± 0.01) for other breeds [29,46,51,54,55]. Apart from the breed differences, the direction of selection regarding these traits [51] could be responsible for such a wide range of correlations across breeds. However, the near absence of correlation in PROD30 males indicates that the genetic basis for both BFT and MS is somewhat independent of each other. Therefore, a selection for higher MS would not unexpectedly affect the breeding goals for lower BFT in Hanwoo cattle. At the same time, to obtain desirable genetic progress, all these traits should be included and properly weighed in a selection index.

Our estimates suggest that the genetic parameters, i.e., h^2^ and r_g_, of PROD30 males for all carcass traits were broadly similar to those reported by previous studies. However, most of the previous reports were based on PROD24 males. Such similarities could be because both these populations share common genetics through common sires, and thus there is a possible common genetic base for traits. Although parameters were somewhat similar across studies, they do not necessarily indicate that the distribution of traits would be similar as well. In fact, our study showed lower CVs (more homogeneity in traits) compared to Park et al. [9], especially in terms of MS, where a CV of 51.0% was reported for PROD24 males. A possible explanation for such variation is that either the population is inherently less variable for such traits, or that more sample homogeneity occurs due to extended feeding periods. 

However, the slight difference in parameter estimates among reports could be for multiple reasons. First, the genetic variance of populations under consideration could vary between studies. It is known that heritability can change at different periods, even in the same breed or population, based on the extent of external interventions such as artificial selection. Second, the difference in sample size and analysis methods could partition the additive genetic variance differently for similar traits among populations, thus generating different genetic variances [11,13]. The Hanwoo studies by Choi et al. [14] and Park et al. [9] provide some support for this suggestion, as the former indicated model differences led to a difference in estimates, and the latter showed that similarities in modeling methods used led to parameter estimate similarities. In addition, our estimated variance parameters were also much higher than those of Choi et al. [14]. However, there are several potential explanations for differing heritability estimates for carcass traits. For example, different data structures were used in the studies, including the relationship between phenotypes measured on the same individual and the number of individuals with phenotypes; different pedigree structures were used in the current study including full- and half-sibling sizes, and the presence of a registration system in parentage errors; these parameters created a different pedigree depth.

In this study, we also demonstrated genetic trends in carcass trait EBVs since 2005. Generally, CWT, EMA, and MS are subjected to positive selection in PROD24 males, whereas BFT is negatively selected because lean meat is targeted [9,14]. For CWT, we observed some consistent genetic improvement over the years, except for 2009, for which the reason remains unclear. One reason for this might be the use of proven bulls which were either genetically relatively inferior or inaccurately ranked at that time due to certain circumstances. For other traits, however, we observed less fluctuation in animals’ EBVs in that particular period, which could be due to their relatively weaker genetic associations with CWT. Overall, we observed desirable changes in all carcass traits since 2010. Provided that no selection is currently conducted on PROD30 animals, these improvements instead pinpoint the selection improvements in the PROD24 population where proven bulls are selected through rigorous screening. Many of those proven bulls are then used for the breeding of PROD30 animals. 

We already mentioned that a ssGBLUP-based GS is ongoing with the PROD24 population [9]. In this PROD30 population study, we showed a comparable genetic potential for heritability of traits and genetic correlations between traits as reported by many earlier reports in PROD24. Considering the present limitations of the PGT program, our next step would be to search for proper methods to incorporate PROD30 in the national Hanwoo evaluation alongside PROD24. As of now, there is no research performed in this regard. Therefore, further in-depth studies on how to incorporate such information into the existing PGT framework are mandatory. One approach would be to pursue a separate evaluation of each dataset using similar or different statistical models under the multiple-trait ssGBLUP. A second approach could be a combined evaluation of both datasets using similar or different statistical models for the multiple-trait ssGBLUP analysis. In this approach, the PROD30 traits would be treated as different genetically correlated traits with PROD24. Although a covariance structure between traits would be absent in this approach, due to each set of progenies having either PROD24 records or PROD30 carcass records, the solution for sires would essentially be based on many more progenies than the first approach. However, it would be interesting to compare the outcomes of both methods. A third approach could be considering both sets of carcass traits as the same traits and fitting an additional age-related factor as a cross-classified effect or a fixed covariate effect to account for the variation in population due to the age differential. For a proven bull, the first two approaches would derive two sets of breeding values for two different sets of carcass traits per sire, which could give Hanwoo breeders access to further options for judging their cattle. With the third approach, however, there would be only one breeding value estimate for each trait per proven bull. Our further work could investigate the impact of combined evaluation on the ranking of proven bulls. Additionally, it is worth mentioning that there are provisions for genotyping of both animal populations under PGT. With this additional genetic information included in GS, we expect that the evaluation of proven bulls would obtain greater accuracy.

## 5. Conclusions

This was the first study involving males raised under the Korean National Genetic Evaluation program from a 30-month production period. To conclude, we found close agreement in the estimates between the current population (PROD30) and many earlier reports on animals using the PROD24 production system. Heritability estimates of carcass traits were moderate to high. There was evidence of similar genetic correlations among traits that have been frequently reported for PROD24 males. In this study, we demonstrated the genetic potential of the PROD30 population: our results suggest that PROD30 males could be a potential source of newer genetic variations, which could benefit genomic selection using genomic marker information. We proposed a model for the evaluation based on the present dataset. As such, we believe that this study also holds significance in understanding the genetic architecture of the population. The present study could help with making decisions on whether to integrate the PROD30 males into the national level evaluation. We also believe that their integration into the ongoing national Hanwoo evaluation could result in the robust estimation of animal genetic effects, especially under the umbrella of genomic selection.

## Figures and Tables

**Table 1:** Details of 30-month production system of Hanwoo cattle.

Item	Animal Production Type ^1^		
	G1	G2	G3
Total males (%)	4659 (65%)	1252 (30%)	181 (5%)
Years of birth	2005–2017	2012–2017	2016–2017
Total batches	25	12	3
Animal batches per year			
first batch	January–April	April–August	January–April
second batch	August–November	October–February	August–November
Total raising location ^2^	7	58	5
Number of sires	553	307	39
Number of dams	4055	1163	173
Slaughter age (month)	27.1–32.9	27.1–32.9	28.7–31.8
Total slaughterhouses	27	31	2

^1^ G1, males produced under the performance-test program but not selected for progeny testing; G2, males produced under progeny test program but not used for proven bull evaluation; G3, animals produced for other purposes. ^2^ Raising locations indicate the various designated provincial breeding centers used for raising G1 and G3 males, and various country-wide progeny-test farms used for raising G2 males since birth.

**Table 2:** Descriptive summary ^1^ on carcass traits of Hanwoo males.

Trait	N	Mean	SD	Min	Max	CV
CWT (kg)	6092	447.00	48.24	220.00	650.00	10.79
EMA (cm^2^)	6092	93.75	11.31	45.00	151.00	12.06
BFT (mm)	6092	12.80	4.73	1.00	36.00	36.93
MS (1–9)	6092	4.89	1.88	1	9	38.38

^1^ CWT, carcass weight; EMA, eye-muscle area; BFT, backfat thickness; MS, marbling score; N, total sample size; SD, standard deviation; Min, minimum value; Max, maximum value; CV, coefficient of variation.

**Table 3:** Estimates of additive genetic (A) and phenotypic (P) variances, and covariances (above diagonal—additive genetic; below diagonal—phenotypic) of carcass traits using multiple-trait animal model in Hanwoo males ^1^.

Trait	CWT	EMA	BFT	MS	CV_g_
CWT	641.24 (A) 1829.64 (P)	82.77	1.20	7.70	0.06
EMA	201.64	45.36 (A) 104.80 (P)	−5.47	4.34	0.07
BFT	48.92	−0.82	9.96 (A) 20.81 (P)	−0.38	0.25
MS	12.88	5.73	0.77	1.78 (A) 3.18 (P)	0.27

^1^ CWT, carcass weight; EMA, eye-muscle area; BFT, backfat thickness; MS, marbling score; CV_g_, genetic coefficient of variation.

**Table 4:** Heritability estimates (diagonals), genetic correlation (above diagonals) and phenotypic correlation estimates (below diagonals) among carcass traits of Hanwoo males.

Trait ^1^	CWT	EMA	BFT	MS
CWT	0.35 ± 0.04	0.49 ± 0.07	0.02 ± 0.09	0.23 ± 0.08
EMA	0.46 ± 0.01	0.43 ± 0.05	−0.26 ± 0.08	0.48 ± 0.07
BFT	0.25 ± 0.02	−0.02 ± 0.02	0.48 ± 0.05	−0.09 ± 0.08
MS	0.17 ± 0.02	0.31 ± 0.01	0.09 ± 0.02	0.56 ± 0.05

^1^ CWT, carcass weight; EMA, eye-muscle area; BFT, backfat thickness; MS, marbling score.

## Data Availability

Data sharing not applicable.

## Supplementary Materials

The following are available online at https://www.mdpi.com/article/10.3390/ani11061792/s1. Table S1. Details of model terms, degrees of freedom, model residual sum of squares (RSS), and Akaike information criterion (AIC) for carcass weight in Hanwoo cattle; Table S2. Details of model terms, degrees of freedom, model residual sum of squares (RSS), Akaike information criterion (AIC) for eye-muscle area in Hanwoo cattle; Table S3. Details of model terms, degrees of freedom, model residual sum of squares (RSS), and Akaike information criterion (AIC) for backfat thickness in Hanwoo cattle; Table S4. Details of model terms, degrees of freedom, model residual sum of squares (RSS), and Akaike information criterion (AIC) for marbling score in Hanwoo cattle.
