# Supplementary material for: Genetic Analysis of Major Carcass Traits of Korean Hanwoo Males Raised for Thirty Months
Source: Animals (Basel). 2021 Jun 15;11(6):1792. doi: 10.3390/ani11061792 (PMC8232619; doi:10.3390/ani11061792)
Supplement: Supplementary file 1 [file animals-11-01792-s001.zip › animals-1208438-supplementary.pdf]

animals

Table S1. Details of model terms, degrees of freedom, model residual sum of squares (RSS), and Akaike Information Criterion (AIC) for carcass weight in Hanwoo cattle[1]

Label	Factor term	DF	RSS	AIC
M1	Intercept	2	14238605.0	64556.0
M2	Intercept; Type	4	14230856.0	64556.7
M3	Intercept; Type; Batch	30	12460396.0	63799.2
M4	Intercept; Type; Batch; RaisedLoc	93	11824460.0	63606.0
M5	Intercept; Type; Batch; RaisedLoc; SlgH	132	11636086.0	63586.1
M6	Intercept; Type; Batch; RaisedLoc;SlgH; SlgAge (c)	133	11465504.0	63498.2
M7	Intercept; Type*Batch*RaisedLoc; SlgH; SlgAge (c)	360	10373846.0	63342.5
M8	Intercept; Type*Batch*RaisedLoc; SlgH (r); SlgAge (c)	326	10419070.0	60926.5
M9	Intercept; Type*Batch*RaisedLoc; SlgH*SlgD; SlgAge (c)	1173	7743978.0	63187.1
M10	Intercept; Type*Batch*RaisedLoc; SlgH*SlgD (r); SlgAge (c)	326	8756221.0	60737.5
M11[2]	Intercept; Type*BirthYS*RaisedLoc; SlgH*SlgD (r); SlgAge (c)	516	8442546.0	59208.8

[1] Intercept, model intercept term; Type, animal type; Batch, batch number of animals; BirthYS, birth year and season combined; RaisedLoc, raising location of the animal until slaughter; SlgH, slaughterhouse; SlgD, slaughter date; SlgAge, slaughter age of the animal; DF, degrees of freedom of the model; terms with ‘r’ and ‘c’ letters within parentheses indicate both random effect and covariate effect, whereas the absence of ‘r’ and ‘c’ with term indicates a fixed effect.

[2] The best model having the lowest RSS and AIC.

Table S2. Details of model terms, degrees of freedom, model residual sum of squares (RSS), and Akaike Information Criterion (AIC) for eye-muscle area in Hanwoo cattle[1]

Label	Factor term	DF	RSS	AIC
M1	Intercept	2	787287.5	46916.0
M2	Intercept; Type	4	785681.1	46907.6
M3	Intercept; Type; Batch	30	687621.6	46147.3
M4	Intercept; Type; Batch; RaisedLoc	93	653682.7	45964.9
M5	Intercept; Type; Batch; RaisedLoc; SlgH	132	639978.9	45913.8
M6	Intercept; Type; Batch; RaisedLoc; SlgH; SlgAge (c)	133	639734.2	45913.5
M7	Intercept; Type*Batch*RaisedLoc; SlgH; SlgAge (c)	360	584437.5	45816.7
M8	Intercept; Type*Batch*RaisedLoc; SlgH (r); SlgAge (c)	326	589548.7	44324.0
M9	Intercept; Type*Batch*RaisedLoc; SlgH*SlgD; SlgAge (c)	1173	443624.9	45763.0
M10	Intercept; Type*Batch*RaisedLoc; SlgH*SlgD (r); SlgAge (c)	326	499293.0	44123.3
M11[2]	Intercept; Type*BirthYS*RaisedLoc; SlgH*SlgD (r); SlgAge (c)	516	480778.5	43186.1

[1] Intercept, model intercept term; Type, animal type; Batch, batch number of animals; BirthYS, birth year and season combined; RaisedLoc, raising location of the animal until slaughter; SlgH, slaughterhouse; SlgD, slaughter date; SlgAge, slaughter age of the animal; DF, degrees of freedom of the model; terms with ‘r’ and ‘c’ letters within parentheses indicate both random effect and covariate effect, whereas the absence of ‘r’ and ‘c’ with term indicates a fixed effect.

[2] The best model having the lowest RSS and AIC.

Table S3. Details of model terms, degrees of freedom, model residual sum of squares (RSS), and Akaike Information Criterion (AIC) for backfat thickness in Hanwoo cattle[1]

Label	Factor term	DF	RSS	AIC
M1	Intercept	2	136254.5	36228.5
M2	Intercept; Type	4	134369.3	36147.6
M3	Intercept; Type; Batch	30	127581.0	35883.7
M4	Intercept; Type; Batch; RaisedLoc	93	122864.0	35780.2
M5	Intercept; Type; Batch; RaisedLoc; SlgH	132	121483.8	35789.3
M6	Intercept; Type; Batch; RaisedLoc; SlgH; SlgAge (c)	133	120780.8	35756.0
M7	Intercept; Type*Batch*RaisedLoc; SlgH; SlgAge (c)	360	111739.4	35735.9
M8	Intercept; Type*Batch*RaisedLoc; SlgH (r); SlgAge (c)	326	112161.7	34770.0
M9	Intercept; Type*Batch*RaisedLoc; SlgH*SlgD; SlgAge (c)	1173	89083.4	35981.2
M10	Intercept; Type*Batch*RaisedLoc; SlgH*SlgD (r); SlgAge (c)	326	101849.1	34705.5
M11[2]	Intercept; Type*BirthYS*RaisedLoc; SlgH*SlgD (r); SlgAge (c)	516	99295.6	34150.9

[1] Intercept model intercept term; Type animal type; Batch batch number of animals; BirthYS birth year and season combined; RaisedLoc raising location of the animal until slaughter; SlgH slaughterhouse; SlgD slaughter date; SlgAge slaughter age of the animal; DF degrees of freedom of the model; terms with 'r' and 'c' letters within parentheses indicate both random effect and covariate effect whereas the absence of 'r' and 'c' with term indicates a fixed effect.

[2] The best model having the lowest RSS and AIC.

Table S4. Details of model terms degrees of freedom model residual sum of squares (RSS), and Akaike Information Criterion (AIC) for marbling score in Hanwoo cattle[1]

Label	Factor term	DF	RSS	AIC
M1	Intercept	2	21425.5	24953.7
M2	Intercept; Type	4	20147.7	24583.1
M3	Intercept; Type; Batch	30	19480.5	24429.9
M4	Intercept; Type; Batch; RaisedLoc	93	18558.7	24260.6
M5	Intercept; Type; Batch; RaisedLoc; SlgH	132	18205.9	24221.7
M6	Intercept; Type; Batch; RaisedLoc; SlgH; SlgAge (c)	133	18195.0	24220.0
M7	Intercept; Type*Batch*RaisedLoc; SlgH; SlgAge (c)	360	17056.6	24280.4
M8	Intercept; Type*Batch*RaisedLoc; SlgH (r); SlgAge (c)	326	17154.5	23923.6
M9	Intercept; Type*Batch*RaisedLoc; SlgH*SlgD; SlgAge (c)	1173	13506.1	24484.6
M10	Intercept; Type*Batch*RaisedLoc; SlgH*SlgD (r); SlgAge (c)	326	15484.1	23851.1
M11[2]	Intercept; Type*BirthYS*RaisedLoc; SlgH*SlgD (r); SlgAge (c)	516	14957.8	23605.5

[1] Intercept model intercept term; Type animal type; Batch batch number of animals; BirthYS birth year and season combined; RaisedLoc raising location of the animal until slaughter; SlgH slaughterhouse; SlgD slaughter date; SlgAge slaughter age of the animal; DF degrees of freedom of the model; terms with 'r' and 'c' letters within parentheses indicate both random effect and covariate effect whereas the absence of 'r' and 'c' with term indicates a fixed effect.

[2] The best model having the lowest RSS and AIC.
